# Supplementary material for: Measuring group fairness in community detection
Source: PLoS One. 2025 Nov 11;20(11):e0336212. doi: 10.1371/journal.pone.0336212 (PMC12604808; doi:10.1371/journal.pone.0336212)
Supplement: S1 File — (PDF) [file pone.0336212.s001.pdf]

Supporting Information for "Measuring Group Fairness in Community Detection"

Elze de Vink, Frank W. Takes, Akrati Saxena

Appendix 1: Community Detection Method Parameters

Table S1 provides details on the implementation of various community detection methods and the parameters used in our analysis. If a method is implemented using the CDlib library, the corresponding function name is provided; otherwise, the implementation details are given.

Appendix 2: Robustness Analysis

We evaluate the robustness of the fairness metric Φ with respect to the community mapping procedure. In the proposed method, communities are matched using Jaccard similarity, and ties are broken uniformly at random. To test robustness, we compare this random tie-breaking with two alternative deterministic strategies:

1. **Map Larger Community First:** In this approach, ties are resolved by prioritizing the larger predicted community. If the predicted communities are of equal size, the larger ground-truth community is selected. Finally, if the similarity score and the sizes of both the ground-truth and predicted communities are identical, the tie is broken by selecting the first community according to the ground-truth label order. The resulting fairness score is denoted by Φ_1.

2. **Map Smaller Community First:** This approach follows the same procedure as described above, except that ties are resolved by prioritizing the smaller predicted community. If the predicted communities are of equal size, the smaller ground-truth community is selected. In cases where similarity scores and both community sizes are identical, the tie is broken by choosing the first community according to the ground-truth label order. The resulting fairness score is denoted by Φ_2.

We compute $\Delta(\Phi, \Phi_1)$ and $\Delta(\Phi, \Phi_2)$, i.e., the differences between the fairness score obtained with random tie-breaking and those obtained using the alternative strategies. Results are reported in Table S2 for the LFR network dataset with mixing parameter $\mu = 0.4$ (refer to Table 3 in the paper), considering $\Phi^{FCCN}_{\text{size}}$, Φ^{F1}_{size}, and $\Phi^{FCCE}_{\text{size}}$. The results indicate that the choice of tie-breaking strategy has a negligible impact as the Δ values are either zero or very close to zero across all cases. This demonstrates that the proposed fairness metric remains stable and robust across different mapping strategies.

Method	Implementation	Parameters
CNM	greedy_modularity	-
Combo	pycombo	weight: "weight"; max_coms: None; modularity_resolution: 1; num_split_attempts: 0; start_separate: False; treat_as_modularity: False; random_seed: 42
Leiden	leiden	initial_membership: None; weights: None
Louvain	louvain	weight: "weight"; resolution: 1; randomize: None
Paris	paris	-
RB-C	rb_pots	initial_membership: None; weights: None; resolution_parameter: 1
RB-ER	rber_pots	initial_membership: None; weights: None; node_sizes: None; resolution_parameter: 1
Significance	significance_coms	initial_membership: None; node_sizes: None
Eigenvector	eigenvector	-
RSC-K	r_spectral_clustering	n_clusters: No. of ground truth communities; method: "regularized_with_kmeans"; percentile: None
RSC-SSE	r_spectral_clustering	n_clusters: No. of ground truth communities; method: "sklearn_spectral_embedding"; percentile: None
RSC-V	r_spectral_clustering	n_clusters: No. of ground truth communities; method: "vanilla"; percentile: None
Spectral	spectral	kmax: No. of ground truth communities; projection_on_smaller_class: True; scaler: None
Deepwalk	Self-implemented	n_clusters: No. of ground truth communities; dimensions: 128; walk_length: 80; num_walks: 10
Fairwalk	Author's code [1]	n_clusters: No. of ground truth communities; dimensions: 128; walk_length: 80; num_walks: 10
Node2Vec	Author's code [2]	n_clusters: No. of ground truth communities; dimensions: 128; walk_length: 80; num_walks: 10
Infomap	infomap	flags: " "
Spinglass	spinglass	spins: 25
Walktrap	walktrap	-
Fluid	async_fluid	k: Number of ground truth coms
Label Propagation	label_propagation	-
EM	em	k: Number of ground truth coms
SBM	sbm_dl	-
SBM-Nested	sbm_dl_nested	-

Table S1. Implementation details and parameter settings for community detection methods. If a method is implemented using the CDlib library, then its function name is provided.

	$\Delta(\Phi, \Phi1)$			$\Delta(\Phi, \Phi2)$		
CDMs	Φ^{FCCN}_{size}-$\Phi1^{FCCN}_{size}$	Φ^{F1}_{size}-$\Phi1^{F1}_{size}$	Φ^{FCCE}_{size}-$\Phi1^{FCCE}_{size}$	Φ^{FCCN}_{size}-$\Phi2^{FCCN}_{size}$	Φ^{F1}_{size}-$\Phi2^{F1}_{size}$	Φ^{FCCE}_{size}-$\Phi2^{FCCE}_{size}$
CNM	0	0	0	0	0	0
Combo	0	0	2.22e-17	0	0	2.22e-17
Leiden	0	0	0	0	0	0
Louvain	0	0	0	0	0	0
Paris	0	0	0	0	0	0
RB-C	0	0	-2.22e-17	0	0	-2.22e-17
RB-ER	2.22e-17	0	0	2.22e-17	0	0
Significance	0	0	0	0	0	0
Eigenvector	0	0	0	0	0	0
RSC-K	0	0	-3.50e-04	0	0	-3.50e-04
RSC-SSE	0	0	-1.60e-04	0	0	-1.60e-04
RSC-V	0	0	0	0	0	0
Spectral	0	0	0	0	0	0
Deepwalk	1.66e-17	3.33e-17	2.77e-17	1.66e-17	3.33e-17	2.77e-17
Fairwalk	0	0	0	0	0	0
Node2Vec	4.15e-05	2.68e-05	3.20e-05	4.15e-05	2.68e-05	3.20e-05
Infomap	0	0	0	0	0	0
Spinglass	0	0	0	0	0	0
Walktrap	0	0	0	0	0	0
Fluid	0	0	0	0	0	0
Label Propagation	0	0	0	0	0	0
EM	-1.31e-05	-1.10e-04	-7.47e-06	1.85e-04	2.31e-04	5.25e-06
SBM	0	6.66e-17	-1.94e-06	0	6.66e-17	-1.94e-06
SBM - Nested	0	0	4.44e-17	0	0	4.44e-17

Table S2. Comparison of fairness scores obtained using random tie-breaking and alternative mapping strategies (larger-first and smaller-first) on the LFR network with $\mu = 0.4$. The small Δ values demonstrate that tie-breaking has negligible effect on the metric.

Appendix 3: Comprehensive Results

Here, we provide the complete results for both synthetic and real-world networks.

Detailed results on LFR Networks

Tables S3, S4, and S5 present the results for LFR networks with μ values of 0.2, 0.4, and 0.6, respectively.

Detailed results on ABCD Networks

Tables S6, S7, and S8 present the results for LFR networks with ξ values of 0.2, 0.4, and 0.6, respectively.

Detailed results on HICH-BA Networks

Tables S9 and S10 present the results for MMaj and MMin networks, respectively.

Detailed results on Real-world Networks

Tables S11, S12, and S13 present the results for Polbooks, Football, and Eu-core networks, respectively.

	Community Quality: Evaluation Metrics					Size			Density			Conductance		
Method	NMI	RMI	ARI	PF1	NF1	ϕ^{FCCN}_{size}	ϕ^{F1}_{size}	ϕ^{FCCE}_{size}	ϕ^{FCCN}_{size}	ϕ^{F1}_{size}	ϕ^{FCCE}_{size}	ϕ^{FCCN}_{size}	ϕ^{F1}_{size}	ϕ^{FCCE}_{size}
CNM	0.586	0.846	0.068	0.524	0.041	0.376	0.168	0.373	-0.036	-0.020	-0.036	-0.323	-0.157	-0.320
Combo	0.912	0.988	0.594	0.737	0.299	0.657	0.634	0.657	-0.394	-0.283	-0.394	-0.672	-0.638	-0.672
Leiden	0.917	0.989	0.614	0.738	0.318	0.652	0.632	0.652	-0.409	-0.294	-0.409	-0.664	-0.635	-0.664
Louvain	0.915	**0.990**	0.608	0.732	0.305	0.653	0.630	0.653	-0.425	-0.307	-0.425	-0.663	-0.630	-0.663
Paris	0.713	0.543	0.591	0.718	0.661	-0.043	**0.011**	-0.043	0.072	**0.017**	0.071	**0.021**	**-0.021**	**0.022**
RB-C	0.918	**0.991**	0.621	0.736	0.313	0.653	0.629	0.653	-0.416	-0.300	-0.416	-0.667	-0.633	-0.667
RB-ER	0.943	0.982	0.729	0.795	0.446	0.590	0.559	0.590	-0.411	-0.375	-0.411	-0.610	-0.571	-0.610
Significance	**1.000**	**1.000**	**0.999**	**0.974**	**0.948**	**0.002**	**0.001**	**0.001**	**0.004**	**0.002**	**0.002**	**-0.006**	**-0.003**	**-0.003**
Eigenvector	0.323	0.133	0.020	0.262	0.064	0.316	0.161	0.273	**-0.008**	-0.023	**0.002**	-0.307	-0.171	-0.259
RSC-K	**0.990**	0.967	**0.963**	**0.984**	**0.969**	**0.031**	**0.021**	**0.019**	0.053	0.044	0.047	-0.072	-0.056	-0.052
RSC-SSE	0.976	0.916	0.744	0.950	0.858	**0.039**	0.056	-0.110	0.159	0.169	0.151	-0.211	-0.225	-0.084
RSC-V	**1.000**	**1.000**	**1.000**	**1.000**	**1.000**	**0.000**	**0.000**	**0.000**	**0.000**	**0.000**	**0.000**	**-0.000**	**-0.000**	**-0.000**
Spectral	0.120	0.281	0.005	0.389	0.004	0.097	0.035	0.096	**-0.010**	**0.005**	**-0.010**	**-0.068**	**-0.028**	-0.068
Deepwalk	0.984	0.944	0.905	0.905	0.762	0.151	0.190	0.088	-0.072	-0.071	-0.059	-0.222	-0.260	-0.171
Fairwalk	0.982	0.937	0.903	0.897	0.738	0.158	0.205	0.076	-0.070	-0.070	-0.040	-0.234	-0.276	-0.171
Node2Vec	0.982	0.936	0.850	0.914	0.768	0.149	0.182	0.089	-0.060	-0.049	-0.040	-0.239	-0.272	-0.194
Infomap	**1.000**	**1.000**	**1.000**	**1.000**	**1.000**	**0.000**	**0.000**	**0.000**	**0.000**	**0.000**	**0.000**	**-0.000**	**-0.000**	**-0.000**
Spinglass	0.727	0.964	0.179	0.326	0.030	0.501	0.214	0.501	-0.060	-0.018	-0.060	-0.437	-0.172	-0.437
Walktrap	**0.995**	0.986	**0.954**	**0.993**	**0.961**	0.083	0.091	0.083	0.044	0.065	0.044	-0.189	-0.214	-0.188
Fluid	0.982	0.937	0.911	0.903	0.758	0.083	0.144	0.037	-0.092	-0.123	-0.076	-0.093	-0.143	-0.053
Label Propagation	0.982	0.958	0.873	0.966	0.839	0.197	0.153	0.197	-0.185	-0.165	-0.185	-0.150	-0.100	-0.149
EM	0.292	-2.072	0.004	0.125	0.063	-0.088	-0.138	**0.010**	-0.042	-0.043	-0.011	0.135	0.174	**0.015**
SBM	0.973	0.978	0.880	0.875	0.663	0.504	0.564	0.493	-0.443	-0.422	-0.442	-0.527	-0.595	-0.521
SBM - Nested	0.983	0.944	0.872	0.900	0.776	-0.300	-0.198	-0.545	0.059	0.037	0.148	0.253	0.163	0.486

Table S3. Results for LFR network with $\mu = 0.2$

	Community Quality: Evaluation Metrics					Size			Density			Conductance		
Method	NMI	RMI	ARI	PF1	NF1	ϕ_{size}^{FCCN}	ϕ_{size}^{F1}	ϕ_{size}^{FCCE}	ϕ_{size}^{FCCN}	ϕ_{size}^{F1}	ϕ_{size}^{FCCE}	ϕ_{size}^{FCCN}	ϕ_{size}^{F1}	ϕ_{size}^{FCCE}
CNM	0.352	0.594	0.025	0.333	0.012	0.172	**0.030**	0.169	**-0.058**	**-0.007**	-0.057	**-0.097**	**-0.018**	**-0.095**
Combo	0.830	0.934	0.366	0.657	0.173	0.634	0.551	0.634	-0.226	-0.165	-0.226	-0.577	-0.478	-0.577
Leiden	0.862	0.958	0.467	0.652	0.192	0.642	0.561	0.642	-0.223	-0.175	-0.224	-0.595	-0.494	-0.595
Louvain	0.869	**0.966**	0.476	0.652	0.201	0.641	0.559	0.641	-0.226	-0.172	-0.227	-0.594	-0.495	-0.594
Paris	0.539	0.438	0.179	0.383	0.144	0.383	0.299	0.357	0.063	**0.016**	0.063	-0.412	-0.296	-0.389
RB-C	0.863	**0.958**	0.471	0.644	0.199	0.640	0.559	0.640	-0.219	-0.171	-0.219	-0.592	-0.493	-0.592
RB-ER	0.872	**0.960**	0.491	0.625	0.206	0.576	0.457	0.576	-0.269	-0.213	-0.271	-0.507	-0.369	-0.506
Significance	**0.985**	0.007	**0.976**	0.536	0.316	**0.078**	0.048	**0.051**	0.163	0.103	0.107	-0.200	-0.127	-0.134
Eigenvector	0.197	0.178	0.016	0.135	0.010	0.181	**0.037**	0.144	**-0.035**	**-0.009**	**-0.026**	-0.136	**-0.027**	**-0.109**
RSC-K	0.933	0.784	0.451	**0.916**	**0.845**	0.146	0.134	0.107	0.308	0.282	0.283	-0.357	-0.335	-0.309
RSC-SSE	0.791	0.314	0.063	0.766	0.614	0.185	0.197	0.144	0.343	0.362	0.318	-0.433	-0.452	-0.398
RSC-V	**0.990**	**0.972**	**0.982**	**0.982**	**0.981**	**0.021**	**0.032**	**0.007**	0.068	0.056	**0.028**	**-0.075**	**-0.077**	**-0.030**
Spectral	0.096	0.187	0.004	0.336	0.004	**0.091**	**0.024**	**0.091**	**-0.021**	**0.003**	**-0.021**	**-0.062**	**-0.024**	**-0.061**
Deepwalk	0.942	0.820	0.767	0.822	0.588	0.214	0.290	0.119	0.369	0.370	0.377	-0.474	-0.517	-0.424
Fairwalk	0.936	0.801	0.746	0.810	0.570	0.257	0.314	0.176	0.370	0.367	0.373	-0.494	-0.525	-0.450
Node2Vec	0.943	0.824	0.783	0.825	0.588	0.225	0.300	0.131	0.357	0.360	0.364	-0.477	-0.521	-0.426
Infomap	**0.989**	**0.971**	**0.977**	**0.980**	**0.960**	**0.071**	0.076	**0.056**	0.135	0.139	0.108	-0.177	-0.187	-0.144
Spinglass	0.703	0.917	0.179	0.326	0.030	0.473	0.195	0.473	-0.195	-0.072	-0.195	-0.329	-0.118	-0.329
Walktrap	0.943	0.870	0.432	**0.968**	**0.814**	0.324	0.324	0.319	0.477	0.491	0.472	-0.581	-0.588	-0.577
Fluid	0.953	0.861	**0.875**	0.842	**0.708**	0.144	0.204	0.113	0.232	0.149	0.237	-0.325	-0.325	-0.308
Label Propagation	0.880	0.830	0.298	**0.895**	0.547	0.426	0.327	0.423	0.174	0.188	0.165	-0.504	-0.433	-0.497
EM	0.191	-1.974	0.001	0.064	0.045	**0.014**	**-0.034**	**0.031**	**-0.053**	-0.054	**-0.013**	**0.029**	**0.070**	**-0.015**
SBM	**0.955**	0.947	**0.836**	0.855	0.616	0.504	0.560	0.473	**0.007**	**0.013**	**0.023**	-0.572	-0.612	-0.560
SBM - Nested	**0.954**	0.837	0.773	0.794	0.576	-0.221	-0.054	-0.574	0.341	0.316	0.441	**-0.077**	-0.220	0.343

Table S4. Results for LFR network with $\mu = 0.4$

	Community Quality: Evaluation Metrics					Size			Density			Conductance		
Method	NMI	RMI	ARI	PF1	NF1	ϕ^{FCCN}_{size}	ϕ^{F1}_{size}	ϕ^{FCCE}_{size}	ϕ^{FCCN}_{size}	ϕ^{F1}_{size}	ϕ^{FCCE}_{size}	ϕ^{FCCN}_{size}	ϕ^{F1}_{size}	ϕ^{FCCE}_{size}
CNM	0.138	0.161	0.008	0.079	0.012	0.084	**-0.000**	0.084	**-0.019**	**-0.002**	**-0.019**	**-0.042**	**0.002**	**-0.041**
Combo	0.356	0.493	0.042	0.234	0.012	0.269	0.068	0.276	-0.042	**-0.005**	-0.044	-0.160	**-0.045**	-0.162
Leiden	0.611	**0.685**	0.179	0.436	0.071	0.513	0.307	0.525	-0.096	-0.061	-0.107	-0.367	-0.188	-0.370
Louvain	0.623	**0.704**	0.181	0.445	0.072	0.509	0.304	0.521	-0.076	-0.047	-0.086	-0.375	-0.198	-0.378
Paris	0.366	0.031	0.058	0.214	0.092	0.087	0.095	**0.058**	0.300	0.157	0.275	-0.334	-0.211	-0.293
RB-C	0.620	**0.690**	0.204	0.439	0.073	0.526	0.322	0.539	-0.101	-0.060	-0.114	-0.378	-0.199	-0.382
RB-ER	0.624	**0.693**	0.211	0.429	0.075	0.514	0.304	0.527	-0.127	-0.077	-0.140	-0.346	-0.165	-0.350
Significance	**0.858**	0.425	**0.712**	0.135	0.041	0.169	0.176	0.176	0.527	0.471	0.477	-0.554	-0.514	-0.519
Eigenvector	0.096	0.076	0.004	0.069	0.002	0.083	**0.011**	0.063	**-0.009**	**-0.002**	**-0.007**	**-0.054**	**-0.007**	**-0.041**
RSC-K	0.739	0.144	0.142	**0.672**	**0.419**	0.213	0.289	0.155	0.514	0.545	0.474	-0.556	-0.602	-0.498
RSC-SSE	0.411	-0.844	0.016	0.210	0.084	**0.051**	0.135	**0.022**	0.119	0.153	0.083	-0.160	-0.253	-0.105
RSC-V	**0.838**	0.525	**0.703**	**0.569**	**0.530**	0.196	0.316	0.214	0.533	0.423	0.497	-0.573	-0.541	-0.552
Spectral	0.047	0.075	0.002	0.328	0.003	**0.062**	**0.012**	**0.061**	**-0.001**	**0.010**	**-0.001**	**-0.047**	**-0.020**	**-0.046**
Deepwalk	**0.769**	0.304	**0.525**	0.556	**0.389**	0.285	0.353	0.281	0.541	0.529	0.533	-0.599	-0.611	-0.593
Fairwalk	0.751	0.252	0.497	0.512	0.357	0.286	0.354	0.286	0.540	0.514	0.532	-0.599	-0.602	-0.592
Node2Vec	**0.773**	0.316	**0.532**	0.569	**0.403**	0.263	0.333	0.255	0.544	0.533	0.539	-0.597	-0.609	-0.592
Infomap	**0.850**	0.596	**0.715**	**0.673**	**0.617**	0.273	0.281	0.276	0.517	0.483	0.476	-0.579	-0.557	-0.552
Spinglass	0.510	0.631	0.112	0.293	0.027	0.411	0.158	0.423	-0.104	-0.037	-0.111	-0.237	-0.081	-0.240
Walktrap	0.674	**190.865**	0.074	0.191	0.111	**0.076**	0.112	0.094	0.558	0.557	0.558	-0.545	-0.559	-0.553
Fluid	0.706	0.135	0.439	0.425	0.296	0.165	0.290	0.161	0.539	0.438	0.534	-0.567	-0.544	-0.563
Label Propagation	0.000	0.000	0.000	0.020	0.000	**0.038**	**0.001**	**0.038**	**-0.012**	**-0.000**	**-0.012**	**-0.006**	**-0.000**	**-0.006**
EM	0.174	-2.471	0.001	0.051	0.037	**0.018**	**-0.025**	**0.031**	**-0.039**	-0.051	**-0.006**	**0.023**	0.063	**-0.015**
SBM	0.657	0.682	0.027	**0.781**	0.240	0.528	0.511	0.540	0.246	0.181	0.238	-0.576	-0.538	-0.581
SBM - Nested	0.677	0.640	0.028	**0.845**	0.335	0.480	0.481	0.493	0.453	0.400	0.448	-0.633	-0.614	-0.637

Table S5. Results for LFR network with $\mu = 0.6$

	Community Quality: Evaluation Metrics					Size			Density			Conductance		
Method	NMI	RMI	ARI	PF1	NF1	ϕ_{size}^{FCCN}	ϕ_{size}^{F1}	ϕ_{size}^{FCCE}	ϕ_{size}^{FCCN}	ϕ_{size}^{F1}	ϕ_{size}^{FCCE}	ϕ_{size}^{FCCN}	ϕ_{size}^{F1}	ϕ_{size}^{FCCE}
CNM	0.608	0.811	0.075	0.546	0.065	0.180	-0.008	0.177	-0.137	-0.017	-0.134	-0.183	-0.166	-0.180
Combo	0.910	0.978	0.608	0.714	0.281	0.654	0.612	0.654	-0.643	-0.574	-0.643	0.475	0.403	0.475
Leiden	0.915	0.979	0.627	0.717	0.292	0.652	0.613	0.652	-0.646	-0.580	-0.646	0.444	0.385	0.444
Louvain	0.915	0.979	0.631	0.715	0.298	0.648	0.610	0.648	-0.640	-0.576	-0.640	0.428	0.370	0.428
Paris	0.743	0.650	0.645	0.794	0.722	-0.068	-0.022	-0.048	0.050	**0.007**	0.040	-0.077	-0.050	-0.067
RB-C	0.916	0.980	0.632	0.721	0.292	0.649	0.610	0.649	-0.647	-0.581	-0.647	0.433	0.371	0.433
RB-ER	0.891	0.913	0.252	0.767	0.391	0.165	0.087	0.165	-0.391	-0.299	-0.391	-0.274	-0.296	-0.274
Significance	**0.999**	**0.999**	**0.998**	0.969	0.939	**-0.004**	**-0.002**	**-0.002**	**0.002**	**0.001**	**0.001**	**-0.001**	**-0.001**	**-0.001**
Eigenvector	0.286	0.108	0.022	0.176	0.040	0.266	0.091	0.214	-0.173	-0.061	-0.133	0.063	0.020	0.044
RSC-K	0.962	0.886	0.600	**0.976**	**0.963**	-0.175	-0.121	-0.132	0.124	0.088	0.097	-0.106	-0.077	-0.083
RSC-SSE	**1.000**	**0.999**	**0.999**	**1.000**	**1.000**	**0.001**	**-0.000**	**0.001**	**-0.001**	**-0.000**	**-0.001**	**0.000**	**-0.000**	**0.000**
RSC-V	**0.998**	**0.993**	**0.970**	**0.997**	**0.989**	-0.051	-0.070	-0.051	0.036	0.048	0.036	-0.032	-0.040	-0.031
Spectral	0.006	0.009	0.000	0.477	0.004	0.038	**-0.006**	0.038	**-0.014**	0.009	**-0.014**	-0.043	-0.040	-0.043
Deepwalk	0.975	0.926	0.837	0.896	0.722	0.063	0.136	-0.118	-0.180	-0.219	-0.062	0.066	0.110	-0.023
Fairwalk	0.968	0.905	0.805	0.872	0.666	0.080	0.175	-0.129	-0.195	-0.253	-0.061	**0.029**	0.078	-0.062
Node2Vec	0.974	0.922	0.835	0.883	0.696	0.066	0.150	-0.125	-0.172	-0.224	-0.047	0.043	0.087	-0.035
Infomap	**1.000**	**1.000**	**1.000**	**1.000**	**1.000**	**-0.000**	**-0.000**	**-0.000**	**-0.000**	**-0.000**	**-0.000**	**-0.000**	**-0.000**	**-0.000**
Spinglass	0.735	0.936	0.199	0.348	0.033	0.516	0.239	0.516	-0.382	-0.155	-0.383	0.282	0.098	0.283
Walktrap	**1.000**	**1.000**	**1.000**	**1.000**	**1.000**	**0.000**	**0.000**	**0.000**	**-0.000**	**-0.000**	**-0.000**	**0.000**	**0.000**	**0.000**
Fluid	0.975	0.925	0.880	0.877	0.704	0.089	0.181	0.049	-0.184	-0.249	-0.156	0.035	0.078	**0.019**
Label Propagation	0.978	0.945	0.804	0.961	0.835	**0.020**	-0.078	**0.024**	-0.027	0.052	-0.030	-0.102	-0.147	-0.095
EM	0.545	-0.214	0.045	0.218	0.136	-0.183	-0.103	-0.110	0.170	0.078	0.112	-0.186	-0.119	-0.125
SBM	0.964	0.981	0.851	0.843	0.590	0.570	0.615	0.570	-0.618	-0.639	-0.618	0.350	0.425	0.350
SBM - Nested	0.995	0.984	0.964	0.960	0.917	-0.058	-0.024	-0.166	0.031	0.008	0.109	-0.032	**-0.012**	-0.097

Table S6. Results for ABCD network with $\xi = 0.2$

	Community Quality: Evaluation Metrics					Size			Density			Conductance		
Method	NMI	RMI	ARI	PF1	NF1	ϕ_{size}^{FCCN}	ϕ_{size}^{F1}	ϕ_{size}^{FCCE}	ϕ_{size}^{FCCN}	ϕ_{size}^{F1}	ϕ_{size}^{FCCE}	ϕ_{size}^{FCCN}	ϕ_{size}^{F1}	ϕ_{size}^{FCCE}
CNM	0.461	0.701	0.039	0.420	0.028	0.157	**0.001**	0.154	-0.106	**-0.005**	-0.103	-0.192	-0.089	-0.185
Combo	0.878	0.973	0.495	0.646	0.186	0.649	0.572	0.649	-0.613	-0.512	-0.613	0.162	0.041	0.162
Leiden	0.887	0.976	0.528	0.659	0.203	0.655	0.581	0.655	-0.629	-0.532	-0.629	0.179	0.069	0.179
Louvain	0.890	0.976	0.537	0.666	0.212	0.654	0.582	0.654	-0.629	-0.533	-0.629	0.199	0.082	0.199
Paris	0.820	0.540	0.576	0.780	0.762	-0.163	-0.063	-0.122	0.133	0.039	0.106	-0.097	-0.055	-0.103
RB-C	0.889	0.976	0.534	0.662	0.214	0.653	0.581	0.653	-0.627	-0.531	-0.627	0.196	0.075	0.196
RB-ER	0.881	0.946	0.404	0.675	0.262	0.443	0.319	0.443	-0.485	-0.365	-0.485	-0.304	-0.276	-0.304
Significance	**0.998**	**1.000**	**0.992**	0.913	0.840	**-0.013**	**-0.007**	**-0.007**	**0.009**	**0.004**	**0.005**	**0.002**	**0.001**	**0.001**
Eigenvector	0.255	0.177	0.023	0.138	0.019	0.226	0.065	0.178	-0.160	-0.044	-0.122	-0.058	**-0.021**	-0.053
RSC-K	0.937	0.815	0.419	0.953	**0.919**	-0.189	-0.130	-0.164	0.149	0.102	0.132	-0.072	-0.052	-0.073
RSC-SSE	0.957	0.877	0.693	**0.969**	**0.962**	**-0.018**	-0.013	**0.005**	0.021	0.017	**0.002**	**-0.008**	**-0.006**	**-0.007**
RSC-V	**0.998**	**0.994**	**0.984**	**0.997**	**0.989**	**-0.018**	-0.023	**-0.017**	**0.019**	0.022	0.018	-0.021	-0.029	**-0.022**
Spectral	0.103	0.209	0.004	0.412	0.005	0.065	**-0.005**	0.064	-0.037	**0.004**	-0.036	-0.080	-0.042	-0.079
Deepwalk	0.974	0.923	0.861	0.872	0.682	0.125	0.215	-0.052	-0.201	-0.262	-0.074	0.067	0.069	0.089
Fairwalk	0.963	0.893	0.810	0.860	0.647	0.145	0.220	-0.024	-0.205	-0.252	-0.086	0.055	0.064	0.078
Node2Vec	0.974	0.922	0.859	0.878	0.685	0.109	0.196	-0.072	-0.174	-0.234	-0.049	0.094	0.084	0.129
Infomap	**1.000**	**1.000**	**1.000**	**1.000**	**1.000**	**-0.000**	**-0.000**	**-0.000**	**-0.000**	**-0.000**	**-0.000**	**0.000**	**0.000**	**0.000**
Spinglass	0.734	0.936	0.200	0.346	0.034	0.500	0.229	0.501	-0.396	-0.162	-0.396	-0.090	-0.049	-0.090
Walktrap	**0.998**	**0.996**	**0.995**	**0.999**	**0.999**	**-0.003**	**-0.003**	**-0.002**	**0.003**	**0.003**	**0.002**	**-0.002**	**-0.002**	**-0.001**
Fluid	0.966	0.901	0.859	0.860	0.688	0.054	0.166	0.030	-0.120	-0.209	-0.099	0.058	0.064	0.058
Label Propagation	0.906	0.858	0.306	0.938	0.636	0.071	-0.078	0.075	**-0.015**	0.101	**-0.017**	-0.118	-0.106	-0.117
EM	0.434	-0.457	0.017	0.109	0.074	-0.115	-0.065	-0.030	0.115	0.060	0.035	-0.077	-0.054	-0.029
SBM	0.969	0.981	0.875	0.859	0.619	0.538	0.591	0.538	-0.585	-0.613	-0.585	0.116	0.207	0.116
SBM - Nested	**0.995**	**0.985**	**0.980**	**0.960**	0.912	0.056	0.088	0.021	-0.067	-0.100	-0.028	**0.004**	0.037	-0.042

Table S7. Results for ABCD network with $\xi = 0.4$

	Community Quality: Evaluation Metrics					Size			Density			Conductance		
Method	NMI	RMI	ARI	PF1	NF1	ϕ_{size}^{FCCN}	ϕ_{size}^{F1}	ϕ_{size}^{FCCE}	ϕ_{size}^{FCCN}	ϕ_{size}^{F1}	ϕ_{size}^{FCCE}	ϕ_{size}^{FCCN}	ϕ_{size}^{F1}	ϕ_{size}^{FCCE}
CNM	0.290	0.433	0.021	0.244	0.012	0.109	-0.004	0.110	-0.061	**0.010**	-0.064	-0.100	-0.019	-0.094
Combo	0.826	0.942	0.355	0.553	0.110	0.607	0.465	0.607	-0.561	-0.402	-0.561	-0.420	-0.298	-0.421
Leiden	0.851	0.954	0.421	0.578	0.134	0.618	0.490	0.618	-0.583	-0.438	-0.583	-0.392	-0.300	-0.392
Louvain	0.853	**0.955**	0.422	0.587	0.138	0.620	0.499	0.620	-0.587	-0.448	-0.587	-0.384	-0.298	-0.384
Paris	0.588	0.257	0.222	0.449	0.340	**0.024**	0.088	0.028	**-0.023**	-0.087	**-0.023**	-0.100	-0.089	-0.095
RB-C	0.853	0.954	0.425	0.584	0.141	0.622	0.505	0.622	-0.591	-0.453	-0.591	-0.403	-0.317	-0.403
RB-ER	0.849	0.943	0.380	0.603	0.161	0.572	0.438	0.572	-0.559	-0.420	-0.559	-0.447	-0.341	-0.447
Significance	**0.978**	**2.689**	**0.923**	0.590	0.369	-0.104	-0.056	-0.071	0.088	0.047	0.060	0.044	0.024	0.031
Eigenvector	0.144	0.110	0.009	0.084	0.005	0.123	0.021	0.094	-0.093	-0.017	-0.070	-0.069	-0.012	-0.053
RSC-K	0.858	0.616	0.133	**0.878**	**0.809**	-0.167	-0.100	-0.148	0.156	0.102	0.141	**-0.002**	-0.016	**0.000**
RSC-SSE	0.757	0.362	0.100	0.775	0.686	-0.086	**0.003**	-0.048	0.078	-0.015	0.041	0.024	**-0.006**	0.029
RSC-V	**0.984**	**0.962**	**0.958**	**0.981**	**0.979**	**-0.048**	**0.003**	-0.033	**0.038**	**-0.010**	**0.027**	0.015	-0.008	**0.009**
Spectral	0.092	0.181	0.004	0.041	0.000	0.073	**0.003**	0.073	**-0.046**	**-0.002**	-0.046	-0.042	**-0.002**	-0.041
Deepwalk	0.957	0.889	0.847	0.865	0.667	0.109	0.193	0.020	-0.171	-0.231	-0.102	-0.046	-0.082	**0.000**
Fairwalk	0.937	0.838	0.791	0.823	0.602	0.137	0.227	0.026	-0.205	-0.269	-0.125	**0.001**	-0.059	0.063
Node2Vec	0.960	0.895	0.855	**0.867**	0.668	0.107	0.193	**0.013**	-0.166	-0.226	-0.091	**0.003**	-0.038	0.049
Infomap	**0.999**	**0.998**	**0.996**	**0.999**	**0.996**	**0.002**	**0.002**	**0.002**	**-0.002**	**-0.001**	**-0.002**	**0.002**	**0.001**	**0.002**
Spinglass	0.733	0.916	0.203	0.354	0.035	0.488	0.224	0.488	-0.406	-0.170	-0.406	-0.308	-0.128	-0.309
Walktrap	0.872	0.694	0.511	0.828	**0.724**	-0.102	-0.079	-0.065	0.079	0.058	0.050	-0.304	-0.336	-0.321
Fluid	0.901	0.746	0.712	0.739	0.552	**0.005**	0.193	**0.009**	-0.049	-0.236	-0.047	0.077	-0.020	0.086
Label Propagation	0.000	0.000	0.000	0.020	0.000	**0.042**	**0.001**	0.042	**-0.026**	**-0.001**	**-0.026**	-0.020	**-0.000**	-0.020
EM	0.356	-0.505	0.004	0.055	0.045	-0.055	-0.029	**0.000**	0.058	0.026	**0.003**	**0.003**	**-0.003**	**-0.005**
SBM	**0.964**	**0.977**	**0.856**	0.849	0.569	0.548	0.592	0.548	-0.605	-0.624	-0.606	-0.209	-0.229	-0.209
SBM - Nested	**0.982**	0.952	**0.921**	**0.895**	**0.752**	0.121	0.210	**0.019**	-0.161	-0.245	-0.053	0.050	0.057	0.068

Table S8. Results for ABCD network with $\xi = 0.6$

	Community Quality: Evaluation Metrics					Size			Density			Conductance		
Method	NMI	RMI	ARI	PF1	NF1	ϕ_{size}^{FCCN}	ϕ_{size}^{F1}	ϕ_{size}^{FCCE}	ϕ_{size}^{FCCN}	ϕ_{size}^{F1}	ϕ_{size}^{FCCE}	ϕ_{size}^{FCCN}	ϕ_{size}^{F1}	ϕ_{size}^{FCCE}
CNM	0.890	0.892	0.915	0.244	0.057	0.281	0.295	0.277	**-0.037**	**-0.022**	**-0.025**	**0.008**	**-0.012**	**0.022**
Combo	**0.961**	**0.962**	**0.977**	**0.865**	**0.720**	0.107	0.134	0.101	-0.216	-0.256	-0.204	-0.092	-0.081	-0.063
Leiden	**0.956**	**0.960**	**0.975**	0.328	0.109	0.103	0.117	0.100	-0.213	-0.222	-0.203	-0.079	-0.085	-0.059
Louvain	**0.956**	**0.960**	**0.975**	0.332	0.111	0.103	0.117	0.100	-0.213	-0.222	-0.203	-0.079	-0.085	-0.059
Paris	0.681	0.680	0.697	**0.915**	**0.652**	0.248	0.250	0.257	-0.447	-0.448	-0.454	-0.186	-0.184	-0.185
RB-C	**0.956**	**0.961**	**0.975**	0.326	0.108	0.103	0.117	0.100	-0.213	-0.222	-0.203	-0.079	-0.086	-0.059
RB-ER	0.294	0.001	0.051	0.002	0.000	-0.444	-0.443	-0.382	0.352	0.340	0.298	-0.231	-0.197	-0.237
Significance	0.302	0.001	0.020	0.003	0.000	-0.299	-0.334	-0.297	0.382	0.380	0.310	0.059	**0.019**	**-0.009**
Eigenvector	0.672	0.685	0.693	0.323	0.069	0.326	0.267	0.349	-0.346	-0.302	-0.363	-0.117	-0.135	-0.099
RSC-K	0.222	0.207	0.041	0.487	**0.487**	**-0.007**	-0.170	**-0.008**	0.171	0.271	**0.099**	0.108	0.064	0.070
RSC-SSE	0.580	0.577	0.537	**0.711**	**0.594**	0.151	0.125	0.152	-0.090	**-0.041**	-0.123	-0.046	**-0.013**	-0.075
RSC-V	0.508	0.501	0.242	**0.649**	**0.500**	-0.292	-0.345	-0.293	0.139	0.169	0.142	-0.344	-0.356	-0.341
Spectral	0.132	0.129	0.077	**0.759**	0.207	0.125	**0.011**	0.119	**-0.018**	0.053	**-0.014**	-0.303	-0.321	-0.306
Deepwalk	0.517	0.511	0.261	0.528	0.179	0.085	0.240	-0.088	-0.327	-0.401	-0.292	-0.128	-0.044	-0.169
Fairwalk	0.513	0.507	0.263	0.512	0.173	0.104	0.246	**-0.021**	-0.331	-0.389	-0.302	**-0.026**	-0.070	-0.096
Node2Vec	0.519	0.513	0.264	0.554	0.217	**0.042**	0.212	**-0.030**	-0.293	-0.420	-0.358	-0.210	-0.126	-0.297
Infomap	0.669	-0.909	0.725	0.018	0.000	-0.113	**-0.062**	**-0.045**	0.073	**0.038**	**0.033**	-0.068	-0.036	**-0.016**
Spinglass	**0.923**	**0.930**	**0.959**	0.279	0.095	0.045	0.088	0.049	-0.151	-0.202	-0.143	-0.039	-0.066	**-0.016**
Walktrap	0.789	-2.897	0.859	0.026	0.001	**0.018**	**0.015**	**0.023**	**0.000**	**-0.005**	**-0.014**	-0.087	-0.096	-0.091
Fluid	0.616	0.614	0.578	0.502	0.155	0.072	0.337	0.186	-0.267	-0.440	-0.335	**-0.028**	-0.040	**-0.001**
Label Propagation	0.635	0.692	0.473	0.069	0.006	-0.189	-0.251	-0.192	0.110	0.176	0.104	-0.206	-0.179	-0.212
EM	0.044	0.033	0.002	0.157	0.017	0.141	0.141	0.200	-0.119	-0.138	-0.144	**0.025**	**0.012**	0.044
SBM	0.875	0.881	0.889	0.409	0.170	**-0.008**	**0.017**	-0.399	**-0.033**	-0.053	0.329	-0.092	-0.102	-0.153
SBM - Nested	0.880	0.884	0.892	0.434	0.184	**-0.025**	**-0.009**	-0.379	**0.020**	**0.006**	0.394	**-0.031**	**-0.030**	-0.056

Table S9. Results for MMaj HICH-BA network

	Community Quality: Evaluation Metrics					Size			Density			Conductance		
Method	NMI	RMI	ARI	PF1	NF1	ϕ_{size}^{FCCN}	ϕ_{size}^{F1}	ϕ_{size}^{FCCE}	ϕ_{size}^{FCCN}	ϕ_{size}^{F1}	ϕ_{size}^{FCCE}	ϕ_{size}^{FCCN}	ϕ_{size}^{F1}	ϕ_{size}^{FCCE}
CNM	0.204	0.168	0.055	0.016	0.000	-0.088	0.175	**-0.047**	-0.316	-0.286	-0.330	**-0.097**	-0.303	-0.127
Combo	0.388	0.372	0.082	0.584	0.312	-0.371	-0.268	-0.399	**-0.055**	-0.147	**-0.037**	0.225	0.088	0.254
Leiden	0.275	0.253	0.044	0.184	0.024	-0.422	-0.226	-0.429	0.231	-0.135	0.234	0.374	**0.025**	0.380
Louvain	0.272	0.250	0.048	0.195	0.026	-0.411	-0.154	-0.440	0.183	-0.252	0.202	0.371	**0.022**	0.397
Paris	**0.694**	**0.694**	**0.759**	**0.890**	**0.450**	0.328	0.342	0.324	-0.485	-0.456	-0.485	-0.481	-0.474	-0.479
RB-C	0.281	0.260	0.045	0.265	0.050	-0.432	-0.233	-0.459	0.237	-0.179	0.255	0.384	0.123	0.409
RB-ER	0.149	0.003	0.036	0.002	0.000	-0.311	-0.208	**-0.020**	0.292	0.220	0.125	0.248	0.151	**-0.038**
Significance	0.134	0.004	0.003	0.004	0.000	-0.294	-0.332	-0.228	0.307	0.296	0.181	0.322	0.339	0.220
Eigenvector	0.147	0.134	0.021	0.364	0.014	0.199	0.328	0.155	-0.308	-0.260	-0.283	-0.277	-0.305	-0.249
RSC-K	0.462	0.432	0.509	0.634	**0.634**	0.327	0.223	0.183	**0.009**	**0.059**	**0.022**	-0.213	-0.121	-0.110
RSC-SSE	0.546	0.527	0.583	**0.713**	**0.662**	0.225	0.165	0.142	-0.064	**0.016**	**-0.018**	**-0.144**	**-0.026**	**-0.070**
RSC-V	**0.848**	**0.845**	**0.905**	**0.782**	**0.557**	0.113	0.155	0.116	-0.437	-0.443	-0.439	-0.384	-0.406	-0.386
Spectral	0.144	0.139	0.123	**0.876**	0.161	0.472	0.454	0.472	-0.296	-0.283	-0.296	-0.545	-0.533	-0.545
Deepwalk	0.200	0.182	0.037	0.374	0.063	0.162	0.252	-0.196	-0.346	-0.432	-0.319	-0.236	-0.314	**0.010**
Fairwalk	0.244	0.227	0.088	0.418	0.096	0.151	0.240	-0.232	-0.363	-0.419	-0.337	-0.199	-0.275	**0.057**
Node2Vec	0.209	0.191	0.048	0.409	0.090	0.149	0.240	-0.220	-0.324	-0.400	-0.281	-0.181	-0.296	0.089
Infomap	0.285	-0.029	0.203	0.015	0.000	-0.225	-0.137	**-0.102**	0.119	0.067	**0.051**	0.197	0.116	0.088
Spinglass	0.260	0.237	0.048	0.183	0.015	-0.324	**-0.031**	-0.311	**-0.053**	-0.329	-0.060	0.221	-0.132	0.208
Walktrap	0.224	0.003	0.165	0.004	0.000	**-0.067**	**-0.044**	**0.031**	0.139	0.094	**0.005**	0.144	0.099	**0.014**
Fluid	0.214	0.198	0.063	0.169	0.002	**-0.045**	0.241	0.167	-0.436	-0.331	-0.505	-0.165	-0.274	-0.328
Label Propagation	**0.801**	**1.150**	**0.858**	0.082	0.007	**0.041**	**0.034**	**0.057**	-0.058	**-0.044**	-0.110	**-0.070**	-0.051	-0.115
EM	0.024	0.002	-0.035	0.147	0.001	0.295	0.376	0.474	-0.210	-0.238	-0.266	-0.286	-0.342	-0.423
SBM	**0.896**	**0.894**	**0.907**	**0.674**	**0.458**	**-0.011**	**0.000**	-0.375	**0.008**	**-0.001**	0.210	**0.010**	**-0.002**	0.328
SBM - Nested	**0.874**	**0.872**	**0.880**	0.597	0.356	**-0.010**	**-0.001**	-0.360	**0.014**	**0.003**	0.262	**0.007**	**-0.005**	0.303

Table S10. Results for MMin HICH-BA network

	Community Quality: Evaluation Metrics					Size			Density			Conductance		
Method	NMI	RMI	ARI	PF1	NF1	ϕ_{size}^{FCCN}	ϕ_{size}^{F1}	ϕ_{size}^{FCCE}	ϕ_{size}^{FCCN}	ϕ_{size}^{F1}	ϕ_{size}^{FCCE}	ϕ_{size}^{FCCN}	ϕ_{size}^{F1}	ϕ_{size}^{FCCE}
CNM	0.531	0.531	0.638	0.510	0.170	0.348	0.346	0.355	0.348	0.351	0.355	-0.334	-0.333	-0.341
Combo	**0.560**	0.565	**0.657**	0.516	0.310	0.237	0.238	0.154	0.260	0.248	0.166	-0.233	-0.230	-0.150
Leiden	0.552	0.555	0.641	0.518	0.311	**0.229**	**0.233**	**0.139**	0.259	**0.247**	**0.163**	-0.227	**-0.227**	**-0.139**
Louvain	0.537	0.540	0.646	0.428	0.257	0.329	0.315	0.290	0.344	0.320	0.297	-0.319	-0.303	-0.280
Paris	**0.565**	**0.567**	0.591	**0.890**	**0.593**	0.506	0.479	0.511	0.485	0.487	0.497	-0.483	-0.466	-0.490
RB-C	0.552	0.555	0.641	0.518	0.311	**0.229**	**0.233**	**0.139**	0.259	**0.247**	**0.163**	-0.227	**-0.227**	**-0.139**
RB-ER	0.497	0.485	0.495	0.416	0.178	**0.161**	**0.194**	**0.081**	**0.134**	**0.169**	**0.034**	**-0.145**	**-0.178**	**-0.063**
Significance	0.386	**1.168**	0.146	0.142	0.014	**0.010**	**0.055**	**-0.071**	**-0.013**	**0.023**	**-0.116**	**-0.003**	**-0.043**	**0.081**
Eigenvector	0.520	0.506	0.547	0.575	0.192	0.236	0.307	0.323	0.263	0.321	0.326	-0.234	-0.298	-0.310
RSC-K	**0.581**	**0.574**	0.641	0.638	**0.638**	**0.100**	0.252	**0.077**	**0.121**	0.261	**0.093**	**-0.101**	-0.243	**-0.078**
RSC-SSE	0.503	0.492	0.534	**0.677**	0.301	**0.002**	0.241	**-0.030**	**0.036**	0.257	**-0.012**	**-0.012**	-0.235	**0.023**
RSC-V	0.534	0.533	0.633	0.494	0.494	0.309	0.327	0.358	0.309	0.332	0.356	-0.296	-0.315	-0.343
Spectral	**0.631**	**0.640**	**0.699**	**0.915**	**0.610**	0.512	0.492	0.513	0.516	0.487	0.517	-0.497	-0.474	-0.498
Deepwalk	0.497	0.495	0.609	0.580	**0.580**	0.309	0.310	0.358	0.309	0.305	0.356	-0.296	-0.295	-0.343
Fairwalk	0.530	0.529	0.636	**0.663**	0.295	0.311	0.329	0.357	0.300	0.323	0.342	-0.295	-0.314	-0.338
Node2Vec	0.525	0.525	0.639	0.585	**0.585**	0.316	0.316	0.360	0.310	0.310	0.356	-0.301	-0.301	-0.344
Infomap	0.529	0.530	0.631	0.432	0.259	0.322	0.311	0.285	0.342	0.319	0.296	-0.315	-0.300	-0.276
Spinglass	0.478	0.456	0.453	0.411	0.206	0.234	0.250	0.165	0.276	0.277	0.210	-0.236	-0.247	-0.170
Walktrap	0.543	0.544	**0.653**	0.522	0.174	0.348	0.342	0.361	0.348	0.345	0.357	-0.334	-0.329	-0.345
Fluid	0.470	0.464	0.574	0.484	0.484	0.295	0.317	0.326	0.306	0.318	0.349	-0.285	-0.303	-0.320
Label Propagation	0.534	0.518	0.594	0.512	0.171	0.383	0.351	0.356	0.392	0.338	0.351	-0.371	-0.332	-0.340
EM	0.091	0.040	0.025	0.453	0.201	-0.230	**0.093**	-0.359	**-0.168**	**0.122**	-0.327	**0.201**	**-0.097**	0.335
SBM	**0.574**	**0.578**	**0.675**	**0.663**	0.295	0.367	0.347	0.368	0.352	0.346	0.358	-0.348	-0.332	-0.350
SBM - Nested	0.554	0.556	**0.665**	0.500	0.375	0.342	0.320	0.301	0.347	0.321	0.299	-0.329	-0.307	-0.288

Table S11. Results for Polbooks real-world network

	Community Quality: Evaluation Metrics					Size			Density			Conductance		
Method	NMI	RMI	ARI	PF1	NF1	ϕ_{size}^{FCCN}	ϕ_{size}^{F1}	ϕ_{size}^{FCCE}	ϕ_{size}^{FCCN}	ϕ_{size}^{F1}	ϕ_{size}^{FCCE}	ϕ_{size}^{FCCN}	ϕ_{size}^{F1}	ϕ_{size}^{FCCE}
CNM	0.698	0.636	0.474	0.752	0.376	0.576	0.523	0.572	**0.306**	**0.191**	0.302	-0.536	-0.446	-0.532
Combo	0.890	0.845	0.807	**0.920**	**0.767**	0.486	0.488	0.490	0.533	0.494	0.529	-0.553	-0.533	-0.549
Leiden	0.890	0.845	0.807	**0.920**	**0.767**	0.486	0.488	0.490	0.533	0.494	0.529	-0.553	-0.533	-0.549
Louvain	0.890	0.845	0.807	**0.920**	**0.767**	0.486	0.488	0.490	0.533	0.494	0.529	-0.553	-0.533	-0.549
Paris	0.831	0.755	0.747	0.788	**0.788**	**0.197**	**0.239**	**0.276**	0.322	0.295	0.446	**-0.298**	**-0.302**	-0.403
RB-C	0.890	0.845	0.807	**0.920**	**0.767**	0.486	0.488	0.490	0.533	0.494	0.529	-0.553	-0.533	-0.549
RB-ER	0.887	0.839	0.791	0.877	0.731	0.491	0.475	0.492	0.340	0.291	0.329	-0.466	-0.433	-0.458
Significance	**0.930**	**0.902**	0.879	0.782	0.587	**0.181**	**0.156**	**-0.084**	0.375	0.295	**0.120**	**-0.323**	**-0.261**	**-0.032**
Eigenvector	0.699	0.597	0.464	0.719	0.607	0.469	0.437	0.424	**0.297**	**0.234**	**0.260**	-0.416	-0.371	**-0.361**
RSC-K	**0.924**	**0.892**	**0.897**	0.897	0.753	**0.358**	0.357	0.413	0.420	0.399	0.469	-0.429	-0.415	-0.477
RSC-SSE	**0.924**	**0.892**	**0.897**	0.897	0.753	**0.358**	0.357	0.413	0.420	0.399	0.469	-0.429	-0.415	-0.477
RSC-V	**0.924**	**0.892**	**0.897**	0.897	0.753	**0.358**	0.357	0.413	0.420	0.399	0.469	-0.429	-0.415	-0.477
Spectral	0.319	0.344	0.121	0.360	0.060	0.426	**0.175**	0.426	**-0.067**	**-0.023**	**-0.067**	**-0.238**	**-0.089**	**-0.238**
Deepwalk	0.916	0.882	**0.888**	0.881	0.740	**0.358**	0.349	0.413	0.420	0.392	0.469	-0.429	-0.408	-0.477
Fairwalk	**0.917**	**0.884**	**0.893**	0.845	**0.845**	**0.299**	0.292	0.413	0.353	0.331	0.469	-0.365	-0.346	-0.477
Node2Vec	0.916	0.882	**0.888**	0.881	0.740	**0.358**	0.349	0.413	0.420	0.392	0.469	-0.429	-0.408	-0.477
Infomap	0.911	0.872	0.857	0.915	**0.838**	0.412	0.400	0.413	0.478	0.463	0.469	-0.484	-0.471	-0.477
Spinglass	0.875	0.821	0.739	0.875	0.729	0.507	0.484	0.524	0.361	0.285	0.372	-0.486	-0.433	-0.499
Walktrap	0.887	0.843	0.815	**0.917**	0.764	0.486	0.505	0.490	0.533	0.496	0.529	-0.553	-0.545	-0.549
Fluid	0.879	0.819	0.764	0.797	0.669	0.360	**0.248**	**0.184**	**0.093**	**0.077**	**-0.269**	**-0.237**	**-0.156**	**0.083**
Label Propagation	0.870	0.806	0.751	0.871	0.660	0.406	0.414	**0.389**	0.547	0.517	0.546	-0.527	-0.508	-0.517
EM	0.357	0.191	0.062	0.346	0.202	**0.213**	**0.031**	**0.254**	**-0.007**	**-0.103**	**0.060**	**-0.126**	**0.043**	**-0.191**
SBM	0.882	0.838	0.813	**0.917**	0.764	0.486	0.500	0.490	0.533	0.498	0.529	-0.553	-0.544	-0.549
SBM - Nested	0.887	0.843	0.815	**0.917**	0.764	0.486	0.505	0.490	0.533	0.496	0.529	-0.553	-0.545	-0.549

Table S12. Results for Football real-world network

	Community Quality: Evaluation Metrics					Size			Density			Conductance		
Method	NMI	RMI	ARI	PF1	NF1	ϕ_{size}^{FCCN}	ϕ_{size}^{F1}	ϕ_{size}^{FCCE}	ϕ_{size}^{FCCN}	ϕ_{size}^{F1}	ϕ_{size}^{FCCE}	ϕ_{size}^{FCCN}	ϕ_{size}^{F1}	ϕ_{size}^{FCCE}
CNM	0.472	0.351	0.171	0.207	0.051	0.431	0.283	0.372	-0.193	-0.137	-0.316	-0.338	-0.244	-0.234
Combo	0.567	**0.594**	0.298	**0.606**	0.101	0.548	0.451	0.558	-0.252	-0.170	-0.260	-0.474	-0.367	-0.484
Leiden	0.575	0.594	0.298	**0.635**	0.121	0.536	0.436	0.549	-0.230	-0.145	-0.244	-0.476	-0.373	-0.486
Louvain	0.601	**0.621**	0.366	**0.615**	0.117	0.580	0.497	0.598	-0.288	-0.213	-0.307	-0.496	-0.393	-0.512
Paris	0.148	0.174	0.018	0.565	0.027	0.382	**0.225**	0.381	-0.121	-0.063	-0.121	**-0.206**	**-0.150**	-0.204
RB-C	0.601	**0.621**	**0.377**	**0.590**	0.112	0.540	0.463	0.552	-0.235	-0.179	-0.247	-0.486	-0.382	-0.496
RB-ER	0.517	0.275	0.078	0.111	0.014	**0.024**	**0.039**	**0.064**	-0.221	-0.210	-0.288	**-0.174**	**-0.163**	-0.172
Significance	**0.678**	0.166	0.293	0.131	0.017	**0.019**	**0.128**	0.164	**0.080**	0.075	-0.178	**-0.188**	-0.302	-0.300
Eigenvector	0.518	0.430	0.265	0.244	0.068	0.508	0.411	0.460	-0.256	-0.203	-0.324	-0.456	-0.351	-0.406
RSC-K	0.591	0.363	0.100	0.463	**0.177**	**0.011**	0.229	-0.127	0.258	**0.059**	**0.074**	-0.219	-0.400	**-0.127**
RSC-SSE	0.573	0.353	0.151	0.453	0.136	**0.072**	0.275	**-0.040**	**-0.066**	**-0.049**	-0.205	-0.263	-0.439	-0.201
RSC-V	**0.717**	**0.599**	**0.543**	0.408	0.156	0.475	0.485	0.425	-0.155	-0.210	-0.365	-0.551	-0.533	-0.503
Spectral	0.149	0.175	0.017	**0.580**	0.028	0.382	0.231	0.380	**-0.121**	-0.065	**-0.120**	-0.207	**-0.155**	-0.204
Deepwalk	0.652	0.479	0.291	0.500	**0.163**	0.166	0.346	**-0.004**	0.215	**-0.036**	**0.008**	-0.338	-0.454	-0.187
Fairwalk	0.658	0.478	0.249	0.495	**0.175**	0.271	0.397	0.177	-0.137	-0.175	-0.319	-0.417	-0.497	-0.362
Node2Vec	0.651	0.471	0.257	0.477	**0.183**	0.088	0.340	-0.078	0.291	**-0.027**	**0.077**	-0.305	-0.472	**-0.170**
Infomap	0.615	0.465	0.248	0.299	0.109	0.462	0.358	0.387	-0.136	-0.106	-0.345	-0.535	-0.476	-0.463
Spinglass	0.533	0.551	0.236	0.442	0.084	0.505	0.367	0.514	-0.240	-0.153	-0.238	-0.400	-0.288	-0.413
Walktrap	0.581	**0.885**	0.200	0.124	0.021	0.446	0.353	0.436	-0.179	-0.128	-0.386	-0.442	-0.379	-0.397
Fluid	**0.700**	0.569	**0.437**	0.472	**0.167**	0.158	0.408	0.153	-0.239	-0.198	-0.226	-0.352	-0.502	-0.363
Label Propagation	0.159	-0.048	0.012	0.177	0.043	0.337	**0.167**	0.327	-0.123	-0.080	**-0.116**	**-0.162**	**-0.099**	**-0.154**
EM	0.167	-0.282	0.002	0.111	0.060	**0.043**	**-0.038**	**0.026**	-0.131	-0.062	-0.246	**-0.028**	**0.003**	**0.035**
SBM	**0.700**	0.532	**0.453**	0.336	0.132	0.311	0.407	**0.015**	**-0.089**	-0.102	-0.357	-0.430	-0.471	**-0.160**
SBM - Nested	**0.696**	0.552	**0.438**	0.447	0.139	0.353	0.433	0.129	**-0.016**	**-0.061**	-0.247	-0.441	-0.493	-0.208

Table S13. Results for Eu-core real-world network

References

1. Rahman T, Surma B, Backes M, Zhang Y. Fairwalk: towards fair graph embedding. In: Proceedings of the 28th International Joint Conference on Artificial Intelligence. IJCAI'19. AAAI Press; 2019. p. 3289–3295.

2. Grover A, Leskovec J. node2vec: Scalable feature learning for networks. In: Proceedings of the 22nd ACM SIGKDD international conference on Knowledge discovery and data mining; 2016. p. 855–864.
